# Surveillance Post Surgery for Retroperitoneal Soft Tissue Sarcoma

**DOI:** 10.3390/curroncol30030211

**Published:** 2023-02-26

**Authors:** John Whitaker, Carolyn Nessim, Max Almond, Samuel J. Ford

**Affiliations:** 1 Midlands Abdominal and Retroperitoneal Sarcoma Unit (MARSU), Queen Elizabeth Hospital Birmingham, Birmingham B15 2GW, UK; 2 Institute of Applied Health Research, University of Birmingham, Birmingham B15 2TT, UK; 3 Academic Department of Military Surgery and Trauma, Royal Centre for Defence Medicine, Birmingham B15 2SQ, UK; 4 Department of General Surgery, Division of Surgical Oncology, The Ottowa Hospital, 501 Smyth Road, Ottawa, ON K1H 8L6, Canada

**Keywords:** sarcoma, retroperitoneal neoplasms, health services, diagnostic imaging

## Abstract

Complete en bloc surgical resection offers the best opportunity for the cure of primary retroperitoneal sarcomas (RPS). The potential for disease recurrence, in the form of both loco-regional recurrence and distant metastases, underpins the rationale for postoperative surveillance. There is a paucity of high-quality evidence underpinning follow-up for RPS patients, and most practice guidelines draw from expert opinion and evidence from soft tissue sarcomas of the extremities. The available observational retrospective data analysis has failed to demonstrate that high-intensity radiological surveillance improves the overall survival in patients. The lack of a robust evidence base has given rise to variations in approaches to post-operative surveillance strategies adopted by specialist centres managing RPS across the world. More high-quality prospective research is needed and planned to more clearly support surveillance approaches that balance oncologic outcomes, patient-centric care, and health service value. Risk stratification tools exist and are available for use in routine practice. Their use will likely support more individualised post-operative surveillance moving forward. Surveillance will likely be underpinned by serial radiological imaging for the medium term. However, developments in genomics offer hope for biomarkers such as ctDNA to impact patient care positively in the future and further support individualised patient care pathways.

## 1. Background

Retroperitoneal soft tissue sarcomas (RPS) are rare malignant tumours with a broad set of histological subtypes. Currently, the best hope for cure is through complete en bloc surgical resection at primary presentation, incorporating adherent adjacent viscera with the specimen [1]. Through the centralisation of patient care at specialist centres, coupled with enhanced surgical techniques and multidisciplinary care, outcomes for patients with RPS have improved [2,3,4]. Nevertheless, overall 5-year survival across retroperitoneal sarcoma patients still sits around 60% [5,6,7]. The potential for disease recurrence, in the form of both loco-regional recurrence (LR) and distant metastasis (DM), underpins the rationale for post-operative surveillance across the spectrum of surgical oncology. Local recurrence (LR) and distant metastasis (DM) remain an enduring source of disease burden for patients with surgically treated RPS. Different histological subtypes of RPS demonstrate different behaviours of recurrence. A striking example of this is that high-grade liposarcomas (LPS) and leiomyosarcomas (LMS) are more likely to recur with DM within two years of index RPS resection. Whereas lower grade LPS pattern of recurrence favours LR with a risk profile that does not plateau but continues to increase over time (26% and 35% LR rates at 5 and 10 years respectively) [8,9].

Currently, there is a paucity of high-quality evidence underpinning post-operative surveillance for RPS patients, and most practice guidelines draw from expert opinion and evidence from soft tissue sarcomas of the extremities. This is problematic due to the substantial differences in both methods of detecting recurrence and the consequences for patient outcomes [10]. Small observational retrospective data analyses of patients undergoing primary resection for RPS have failed to establish an overall survival benefit for high-intensity radiological surveillance for patients with high or low-grade disease [7]. The lack of a robust evidence base has given rise to variations in approaches to postoperative surveillance strategies adopted by specialist centres managing RPS across the world. This includes both frequency and choice of modality in imaging schedules.

More frequent surveillance strategies can be a substantial burden. They impact health services through costs associated with radiological imaging and health care worker time as well as patients who have to take time away from their own productive economic and social activities. The psychological impact of intensive surveillance may be complex. Some patients experience anxiety and heightened awareness of a cancer diagnosis around the time of surveillance investigations, whilst others find solace in the reassurance of ongoing imaging. The cost-effectiveness and impact on quality of life of postoperative surveillance for primary RPS are yet to be established.

In light of the diversity of RPS subtypes, some centres have adopted a standard protocol for all postoperative patients, regardless of final histological diagnosis and recurrence risk. This pragmatic approach has advantages for standardising implementation within a health service. Other centres adopt a surveillance protocol stratified according to the risk of recurrence derived from the final tumour grade. The European Society for Medical Oncology guidelines is one example advocating for a binary low- vs. high-risk-based stratification [1]. It may also be possible to further individualise post-surgical surveillance using more specific recurrence risk estimates based on patient and tumour-specific characteristics and nomograms [11]. Tools such as the smartphone-based Sarculator app can be used in support of this approach [12]. Adopting a more risk-based follow-up approach has the appeal of being more patient-centric as well as a more just allocation of finite resources to where recurrence risk is greatest. This heterogeneous practice across specialist institutions highlights the contemporary uncertainty around best surveillance practices for post-surgical RPS patients, driven by a lack of clear evidence.

## 2. Learning from Sarcoma of the Extremities

Similar to RPS, extremity soft tissue sarcoma patients undergo routine post-surgical surveillance. There are some data from randomised studies available to extrapolate from and inform management in RPS patients. The Trial for Optimal Surveillance in Sarcomas (TOSS) [13] was a randomised trial conducted at a single tertiary centre in India. It found there was non-inferiority between a chest radiograph surveillance group compared with a computerised tomography (CT) scan surveillance group and between a less intensive (six-monthly) surveillance period and a more intensive (three-monthly) surveillance interval using a two-by-two design. The authors concluded that for the extremity sarcoma population, six-monthly interval imaging with chest radiograph combined with patients trained to conduct self-examination will identify the majority of recurrent disease without compromising patient outcomes. However, the noninferiority of overall survival between the 6- and 3-month follow-up intervals was not conclusive due to sample size limitations. Importantly, almost all (88%) of LRs were noted by patients through self-examination, limiting the generalisability of findings to the RPS patient population suffering impalpable intra-abdominal recurrences [14]. This study did not report patient-reported outcome measures (PROMs) such as quality of life.

The Surveillance After Extremity Tumour Surgery (SAFETY) trial is a multi-centre 2 × 2 factorial randomised controlled trial of patients with resected non-metastatic primary soft tissue sarcoma [15]. In a similar 2 × 2 design to TOSS, they use four different surveillance schedules: chest CT every 3 months or every 6 months for 2 years, or chest radiograph (CXR) every 3 months or every 6 months for 2 years. As its primary outcome, SAFETY will compare overall survival between groups in addition to secondary outcomes of LR and DM-free survival as well as several patient-reported outcome measures, including patient anxiety, satisfaction, quality of life, and cost. However, the results may not be available to inform practice before 2027.

Focusing on patient anxiety through the Fear of Cancer Recurrence Inventory scores as its primary outcome, with other PROMs and oncological secondary outcomes, another ongoing trial (NCT04751409) is evaluating sarcoma surveillance regimes [16]. This study is comparing the impact of follow-up every 3 months over 2 years with CT chest and imaging of the primary site and more limited follow-up every 6 months for 2 years with either CT or x-ray of the chest and imaging of the primary site. Enrolled patients will have completed sarcoma therapy (chemotherapy, radiation therapy, and/or surgery) for disease of the trunk or extremity. This study is estimated to be completed at the end of 2024.

A modelling study using data from a relatively small cohort of extremity STS patients in Australia determined that CT chest alone as a surveillance imaging modality is more cost-effective than magnetic resonance imaging (MRI) alone or in combination with CT chest and computerised tomography positron emission tomography (CTPET) alone [17]. However, cost-effectiveness comparisons of CT chest with plain chest radiograph surveillance regimes favour the latter [18]. Taking a recurrence risk stratification approach further enhances the potential cost benefit for plain chest radiographs in those patients assessed as having a lower risk of recurrence [18].

Whilst conducting rigorous randomised clinical trials studying surveillance regimes for sarcoma patients is difficult, such research is certainly warranted to strengthen the limited current evidence base to inform future best practice recommendations, a fact widely accepted by sarcoma clinicians [19]. Exposing their patients to unnecessary radiation exposure as part of surveillance is a concern of most sarcoma specialists [19].

Currently, decisions for sarcoma patient follow-up regimes may not be evidence-based, with marked variation in the practice of both imaging techniques and intensity of follow-up [19,20,21]. Variations in routine practice between countries have been observed, with CT chest routinely preferred in North America and more equipoise between this and a plain radiograph in Europe and Asia [22]. Whichever choice of surveillance imaging is embarked upon, a baseline investigation shortly after completing treatment of the index primary sarcoma is accepted as necessary to allow future comparison and identification of lesions suspicious of recurrent disease.

## 3. Current RPS Surveillance Recommendations

Earlier detection of LR of RPS within the abdomen through contrast-enhanced CT may allow for a better prospect of resection and avoid morbid sequelae associated with large recurrences, such as gastric compression [23]. CT is the imaging technique recommended by several guidelines-issuing organisations for surveillance of patients treated for soft tissue sarcoma, namely the European Society for Medical Oncology (ESMO), the National Comprehensive Cancer Network (NCNN), and the Transatlantic Australasian Retroperitoneal sarcoma Working Group (TARPSWG). The lungs are one of the most common sites for DM of soft tissue sarcoma. Earlier detection of DM within the thorax may enable potentially curative resection, as well as other local or systemic treatments, prior to the onset of attributable symptoms. For this reason, the thorax is routinely included in surveillance CT protocols.

More intensive follow-up regimes are recommended by these organisations for those RPS patients stratified as having a higher recurrence risk. Closer surveillance intervals, typically every 3 months, are recommended for between 2–3 years following primary resection.

The 2021 US NCNN guidelines stipulate patient assessment at 3–6 months for 2–3 years, followed by every six months until the fifth year and annually thereafter. These patient assessments should be conducted with both physical assessment and radiological imaging by way of CT of the chest, abdomen, and pelvis, with abdominal MRI an acceptable alternative for abdominal recurrence examination [24].

The TARPSWG guidance consists of a consensus document that advocates for lifelong surveillance following RPS resection given the disease’s enduring potential risk of recurrence [25]. This phenomenon of ongoing risk is especially evident for the well-differentiated and grade 2 dedifferentiated LPS subtypes [5]. Additionally, where the disease biology would infer a lower risk of DM, such as some low-grade histological subtypes, a CT scan of the thorax may be considered unnecessary. The working group also highlights the potential utility of nomograms to individualise patient follow-up according to data-driven estimates of personal recurrence risk. Accordingly, they advocate post-operative surveillance combining physical assessment and surgery every 3–6 months for the first five years, followed by an annual indefinite physical and radiological assessment.

The ESMO guidelines do not differentiate recommendations for RPS from other sarcomas [1]. They recommend patients stratified into high and intermediate risk categories receive surveillance imaging every 3–4 months for the first two or three years, followed by six monthly imaging until year five and annual imaging until year ten post-surgery. For patients deemed low risk for recurrence, a less intensive recommendation of six monthly imaging exams for the first five years is followed by annual imaging exams until year ten post-surgery. The recurrence risk may be broadly correlated to the RPS tumour grade, with high-grade disease being followed up on a high recurrence risk pathway. While the guidance does not specify the type of radiological investigation to be performed, a cross-sectional CT of the chest with a CT or MRI of the abdomen is considered acceptable.

## 4. Ongoing Surveillance for Recurrent Disease

Poor patient survival outcomes are associated with recurrent RPS [26,27]. The six-year disease-free survival following resection for locally recurrent disease is approximately one fifth, with overall survival only around a half [27]. The aforementioned consensus documents have no explicit guidance for follow-up regimens for RPS patients following surgical treatment of LR or DM. LR is the most common pattern of disease recurrence in the case of well-differentiated and grade 2 dedifferentiated LPS. There is some evidence that treating both first and second LR RPS with surgical excision is associated with improved overall survival [28]. However, whilst this observed finding from retrospective analyses may be due to a true benefit from surgery, favourable patient selection characteristics will also influence this observation. Given the lack of guidance to the contrary, and for ease of health service delivery, the same surveillance protocols in place for the primary disease may be reasonably adopted following recurrent disease resection.

Data-driven decision-making can use individual patient risk estimates for both recurrence and risk of death, which can again be derived from nomograms after surgery for the first recurrence. These can help stratify patients into higher-risk categories for whom more frequent surveillance strategies may be adopted. Furthermore, the intensity of follow-up may also increase following a second or subsequent surgically resected recurrent disease because the disease-free period is likely to shorten.

High-grade LPS infers a higher risk for both LR and DM compared to LMS, for which DM, primarily in the lung, is the most common manifestation of disease recurrence. A number of patient and tumour factors influence the risk of further disease following pulmonary metastasectomy; these include older age, a disease-free interval of under a year, synchronous disease, histological type, and treatment modality [29]. A greater number of factors is associated with worse overall survival, as good as 64% for only two factors compared to as poor as 3% with five adverse factors present. Other factors, such as the number of metastases and the feasibility of resection, are important [30,31,32]. Such risk modelling can help identify patients at particularly high risk of oncological recurrence. Outside of clear guidance and with a better understanding of PROMs awaited, for patients for whom more intensive surveillance may be considered, a shared decision-making approach with the clinical teams and patients themselves should be adopted to ensure whatever approach is taken is in their best interests.

## 5. Considerations in Sarcoma Predisposing Genetic Syndromes

There are several genetic syndromes associated with an increased risk of RPS. Neurofibromatosis type 1 (NF-1) is a complex condition inherited in an autosomal dominant manner. NF1 tumour suppressor gene germline mutations underlie the disorder. In addition to dermal neurofibromas, patients frequently have cutaneous manifestations such as Lisch nodules and café-au-lait spots. Skeletal abnormalities, brain tumours, and learning disabilities are common, in addition to a predisposition to peripheral nerve tumours, including malignant peripheral nerve sheath tumours (MPNST) [33]. While MPNST can develop spontaneously, it can also develop from a plexiform neurofibroma. The lifetime risk approaches 1 in 10, with incidence highest in the 2nd–3rd decade [34]. Whole-body MRI is commonly used to assess whole-body tumour burden in patients with NF-1 [35,36]. FDG-PET/CT has evidence supporting its use as a non-invasive test to discriminate between benign and malignant plexiform neurofibromas in lesions of concern due to growth or new symptom onset [37].

Constitutional pathological TP53 gene variations lie behind the increased cancer risk in Li-Fraumeni and TP53-related heritable cancer syndromes. They predispose to a large number of malignancies in addition to sarcoma, with a cancer diagnosis almost certain by the seventh decade of life [38]. Routine surveillance protocols of TP53 mutation carriers to detect asymptomatic malignancies early has been shown to offer potential survival benefit when implemented [39]. Whole-body MRI (WBMRI) enables early detection of tumours in carriers, and European guidelines advocate whole-body MRI from the first year of life if the variant is associated with childhood cancers [40]. Other features of carrier surveillance include physical examination, including dermatological examination, biochemistry and haematology investigations, focused MRI of the breast and brain, abdominal ultrasound, and lower gastrointestinal endoscopy [39,40]. Following a diagnosis of sarcoma in this population, the treatment approach should be carefully and pro-actively considered [25]. Specific attention should be given to limiting patient exposure to genotoxic chemotherapy and radiation, both of which can give rise to additional future malignancies [40,41]. Individual patient post-surgical surveillance strategies should be adjusted in light of a different risk profile from the general population and in the context of coordinated multidisciplinary care, despite the lack of specific guidance for post-sarcoma surveillance in these populations.

## 6. Personalised Medicine and Risk Stratification

With the aspiration to achieve data-driven, patient-centred, individualised care, tools to provide actionable estimates of prognosis for patients diagnosed with RPS are a valuable contribution. Nomograms devoted to this patient group have provided a marked improvement in this aspect of patient care over the past few years.

A number of disease-, patient-, and therapy-specific factors are implicated in the risk of adverse oncological events (LR and DM) and overall survival after surgical excision of RPS. The specific histological subtype of RPS tumours, as well as with their grade and size, clear resection margins, and patient age are implicated in patient prognosis [5]. Modelling these factors within an available RPS patient population nomogram can offer patient-specific predictions of survival and disease recurrence. One such prediction model initially developed was the Sarculator, which was able to calculate overall survival and disease-free survival at seven years from the point of primary RPS resection [11]. Other nomograms incorporating additional factors such as location, number of resected organs, and association with radiation are available for predicting LR, DM, and disease-specific death and providing prognostic estimates for 3, 5, and 10-year periods [42]. Such prediction tools are valuable for stratifying risk and determining the most suitable surveillance strategy for individual patients. However, their validation is based on a specific time point of index RPS surgical excision rather than dynamic use throughout the patient’s life course.

In addition to nomograms based on factors present at the time of index resection, prognostic prediction modelling at the time of the first LR from the RPS have also been developed. The prediction tool models disease-free and overall survival at six years. This initiative from the TARPSWG incorporates patient age at the time of the second operation, tumour multifocality and grade, completeness of resection at the second operation, histological subtype, use of chemoradiotherapy at the primary operation, and how many organs were initially resected [27]. This still suffers from the limitation of being a static prognostic estimate and not evolving with the patient during surveillance.

As already mentioned, the risk of LR persists for many years for some histological subtypes, specifically well-differentiated LPS and low-grade dedifferentiated LPS [5]. However, for other subtypes, the residual risk of recurrence after several disease-free years is attenuated. Similarly, treated disease recurrence following initial primary resection will adversely affect the residual recurrence risk. The relative weighting and prognostic impact of variables included during baseline prognostic estimates may change due to their time-dependent nature. Dynamic, rather than static, nomogram risk prediction tools can account for the residual prognostic impact of all these factors at various points during the follow-up period after primary surgical resection [43]. The most recent incarnation of the Sarcoma App-based prediction tool incorporates externally validated nomogram modelling for RPS patients [44]. This allows dynamic estimation of residual 5-year overall survival and 5-year disease-free survival from any month within the first 60 days following primary RPS resection. In general terms, the survival prognosis steadily improves during the surveillance period until and unless a recurrence occurs, at which point there is a substantial reduction. Both final models incorporated time since primary surgery and tumour grade. Additionally, the overall survival model included age, completeness of resection, and recurrence, whereas the disease-free survival model additionally included histological subtype, tumour size, multifocality, and the interaction between time elapsed, size, grade, and multifocality [44].

For informing patient surveillance strategies stratified by risk, a dynamic prognostic modelling tool may allow patients to move between higher and lower risk pathways during their follow-up. This offers an evidence-based method for equitably deploying finite health system resources and minimising unnecessary patient follow-up burden. Residual recurrence risk can be quickly calculated at each follow-up encounter to revisit the most appropriate pathway for ongoing surveillance.

## 7. Optimal Surveillance Frequency

Although intensive short-interval follow-up is advocated for certain post-surgical RPS patients [25], the benefits are worthy of further interrogation. A retrospective analysis of patients who underwent surgical treatment for primary, well-differentiated LPS at a single centre in the US failed to support more intensive follow-up regimes [45]. Because surgical treatment of recurrence of well differentiated LPS may not result in a cure, reserving treatment for situations such as high symptom burden from recurrence can influence clinician decision-making regarding the timing of any further operative intervention [46]. For the majority of the 91 patients followed up across the decade of this retrospective analysis, a surveillance strategy using 4- or 6-month intervals would not have changed patient management in 96% and 91% of cases, respectively, compared to more intensive 3-month intervals [45]. Recurrence occurred in 60% of this cohort. There was no evidence of a positive association between survival benefit and more frequent follow-up in one UK single-centre retrospective analysis [7]. Indeed, for high-grade tumours, the disease-free survival was reduced, and no significant difference in reoperation rates following recurrence was seen [7].

More research is needed into the patient-reported outcomes associated with postoperative surveillance. Surgical treatment for RPS may involve extensive multi-visceral resection. A 1-year prospective cohort study from Milan found a post-operative Clavien Dindo complication grade 3 or higher in one-quarter of patients with a 1.3% mortality rate. Enduring neuropathic pain was found in two fifths of patients, particularly if some psoas muscle was resected. Despite this, there was a non-significant increase in quality of life for patients compared to baseline, with comparable scores to the general local population [47].

A thematic analysis of interviews with patients and families treated for predominantly extremity sarcomas has shed some interesting light on patient experience and perspectives around surveillance [48]. A past sarcoma diagnosis was seen as a long-term mortality threat over which patients had little autonomy. The relationship with surveillance was found to be complex, engendering mixed emotional responses. A cyclical experience of peri-scan anxiety followed by reassurance was akin to a “merry-go-round” [48]. This observed psychological distress associated with imaging following a cancer diagnosis is not unique to sarcoma and is associated with impaired quality of life in other malignancies [49,50]. This again highlights the importance of considering the psychological burden of surveillance in follow-up strategies.

## 8. Looking Ahead

In the absence of a strong evidence base for meaningful patient survival benefit, caution should be exercised before routinely embarking on high-intensity surveillance for all post-surgical RPS patients. Similarly, such a well-intentioned practice may be causing unintended harm through psychological patient burden and overconsumption of limited health service resources.

Contemporary management of recurrent RPS, both LR and DM, often includes a period of further expectant monitoring to establish the biological behaviour of the disease and for the presence of any additional synchronous occult disease to become clear. Interventions are rarely initiated immediately after identifying a possible recurrence, leading to a substantial time lapse between detection and active therapies. Therefore, the intrinsic delay associated with less intensive surveillance protocols for detecting recurrent disease may not lead to observable patient harm.

To address the evidence gap in post-RPS resection surveillance, hope is on the horizon by way of the SARveillance trial [10]. This trial aims to address much of the uncertainty surrounding post-surgical surveillance for patients with retroperitoneal, abdominal, or pelvic soft tissue sarcoma. The intention is to support evidence-based, standardised practice guidelines across specialist sarcoma treatment centres internationally [45,46]. SARveillance has benefited from a collaborative, multinational approach, with stakeholders from several countries forming the trial steering group. A patient-centred approach is strengthened by engagement from a multinational patient advisory group. It is a multicentre trial comparing high and low-intensity post-surgical surveillance at high-volume specialist centres from the UK, Europe, North America, and Australasia. Inclusion criteria include adult patients with surgically complete resection of non-recurrent, non-metastatic, primary soft tissue sarcomas of the retroperitoneum, abdomen, and pelvis. Important exclusion criteria include incomplete resection, metastatic disease, and certain specific histological subtypes, including Ewing’s STS, uterine sarcomas, and desmoplastic small round cell tumours. Following a baseline CT scan, patients will be enrolled and stratified according to the final tumour grade. Within each tumour grade, patients will be randomly assigned in a 1:1 ratio to either high- or low-intensity surveillance. The planned primary study outcomes are quality of life and overall survival, and we aim to recruit 650 patients within 48 months.

Any eligible sarcoma patient who does not wish to participate in the randomised protocol of SARveillance will be able to choose their own level of surveillance intensity as part of a patient preference arm [10]. This is important to not only for maximizing the collection of actionable prospective patient data, but also for highlighting patient reasons for surveillance preference and observing any PROM differences between those randomly assigned to one or the other surveillance pathway. It is anticipated that there will not be a substantial additional cost burden for participating centres since the frequency of surveillance within both arms of the trial is within the known spectrum of routine practice.

The study’s analysis will also include health economics, and prospective data will help to develop and validate a recurrence risk prediction model. If able to deliver on its intentions, SARveillance should be able to establish and drive cost-effective, clinically efficacious, patient-centred standardisation and follow-up protocols for broad application to sarcoma patients globally.

Liquid biopsy is a term for the genomic technologies able to identify, quantify, and profile material derived from tumours to be detected in the peripheral circulation and elsewhere [51]. This novel technology has the potential to be used as prognostic, therapeutic monitoring, and surveillance biomarkers. However, their use in sarcomas lags behind that of other malignancies [52]. Retrospective studies have shown patients with sarcomas have detectable levels of circulating tumour DNA (ctDNA). One study of LMS patients found a positive correlation between higher levels of ctDNA and tumour size and disease progression [53]. However, this nascent field is yet to break into mainstream clinical practice for sarcoma patient surveillance.

## 9. Summary

Ongoing post-operative follow-up for patients with resected retroperitoneal sarcoma is routine internationally, but with substantial variation in practice, reflecting the current paucity of high-quality evidence to guide practice. More high-quality prospective research is needed and planned to more clearly support surveillance approaches that balance oncologic outcomes, patient-centric care, and health service value. Risk stratification tools exist and are available for use in routine practice. Their use will likely support more individualised post-operative surveillance moving forward. For the medium term, surveillance will likely be underpinned by serial radiological imaging. However, developments in genomics offer hope for biomarkers such as ctDNA to impact patient care positively in the future and further support individualised patient care pathways.

## References

[1] Gronchi A., Miah A.B., Dei Tos A.P., Abecassis N., Bajpai J., Bauer S., Biagini R., Bielack S., Blay J.Y., Bolle S. (2021). Soft tissue and visceral sarcomas: ESMO-EURACAN-GENTURIS Clinical Practice Guidelines for diagnosis, treatment and follow-up(☆). Ann. Oncol..

[2] Kalaiselvan R., Malik A.K., Rao R., Wong K., Ali N., Griffin M., Chandrasekar C.R., Fenwick S.F., Poston G.J., Malik H. (2019). Impact of centralization of services on outcomes in a rare tumour: Retroperitoneal sarcomas. Eur. J. Surg. Oncol..

[3] Vos M., Blaauwgeers H.G.T., Ho V.K.Y., van Houdt W.J., van der Hage J.A., Been L.B., Bonenkamp J.J., Bemelmans M.H.A., van Dalen T., Haas R.L. (2019). Increased survival of non low-grade and deep-seated soft tissue sarcoma after surgical management in high-volume hospitals: A nationwide study from the Netherlands. Eur. J. Cancer.

[4] Callegaro D., Raut C.P., Ng D., Strauss D.C., Honoré C., Stoeckle E., Bonvalot S., Haas R.L., Vassos N., Conti L. (2021). Has the Outcome for Patients Who Undergo Resection of Primary Retroperitoneal Sarcoma Changed Over Time? A Study of Time Trends During the Past 15 years. Ann. Surg. Oncol..

[5] Gronchi A., Strauss D.C., Miceli R., Bonvalot S., Swallow C.J., Hohenberger P., Van Coevorden F., Rutkowski P., Callegaro D., Hayes A.J. (2016). Variability in Patterns of Recurrence After Resection of Primary Retroperitoneal Sarcoma (RPS): A Report on 1007 Patients From the Multi-institutional Collaborative RPS Working Group. Ann. Surg..

[6] Chouliaras K., Senehi R., Ethun C.G., Poultsides G., Tran T., Grignol V., Gamblin T.C., Roggin K.K., Tseng J., Fields R.C. (2019). Recurrence patterns after resection of retroperitoneal sarcomas: An eight-institution study from the US Sarcoma Collaborative. J. Surg. Oncol..

[7] Glasbey J.C., Bundred J., Tyler R., Hunt J., Tattersall H., Gourevitch D., Almond L.M., Desai A.D., Ford S.J. (2021). The impact of postoperative radiological surveillance intensity on disease free and overall survival from primary retroperitoneal, abdominal and pelvic soft-tissue sarcoma. Eur. J. Surg. Oncol..

[8] Trojani M., Contesso G., Coindre J.M., Rouesse J., Bui N.B., de Mascarel A., Goussot J.F., David M., Bonichon F., Lagarde C. (1984). Soft-tissue sarcomas of adults; study of pathological prognostic variables and definition of a histopathological grading system. Int. J. Cancer.

[9] De Sanctis R., Giordano L., Colombo C., De Paoli A., Navarria P., Sangalli C., Buonadonna A., Sanfilippo R., Bertola G., Fiore M. (2017). Long-term Follow-up and Post-relapse Outcome of Patients with Localized Retroperitoneal Sarcoma Treated in the Italian Sarcoma Group-Soft Tissue Sarcoma (ISG-STS) Protocol 0303. Ann. Surg. Oncol..

[10] Baia M., Ford S.J., Dumitra S., Samà L., Naumann D.N., Spolverato G., Callegaro D. Follow-up of patients with retroperitoneal sarcoma. Eur. J. Surg. Oncol..

[11] Gronchi A., Miceli R., Shurell E., Eilber F.C., Eilber F.R., Anaya D.A., Kattan M.W., Honoré C., Lev D.C., Colombo C. (2013). Outcome prediction in primary resected retroperitoneal soft tissue sarcoma: Histology-specific overall survival and disease-free survival nomograms built on major sarcoma center data sets. J. Clin. Oncol..

[12] Callegaro D., Miceli R., Bonvalot S., Ferguson P., Strauss D.C., Levy A., Griffin A., Hayes A.J., Stacchiotti S., Pechoux C.L. (2016). Development and external validation of two nomograms to predict overall survival and occurrence of distant metastases in adults after surgical resection of localised soft-tissue sarcomas of the extremities: A retrospective analysis. Lancet Oncol..

[13] Puri A., Gulia A., Hawaldar R., Ranganathan P., Badwe R.A. (2014). Does intensity of surveillance affect survival after surgery for sarcomas? Results of a randomized noninferiority trial. Clin. Orthop. Relat. Res..

[14] Puri A., Ranganathan P., Gulia A., Crasto S., Hawaldar R., Badwe R.A. (2018). Does a less intensive surveillance protocol affect the survival of patients after treatment of a sarcoma of the limb?. Bone Jt. J..

[15] SAFETY Investigators (2019). The Surveillance After Extremity Tumor Surgery (SAFETY) trial: Protocol for a pilot study to determine the feasibility of a multi-centre randomised controlled trial. BMJ Open.

[16] Evaluating the Impact of Limited Compared With Intense Post-Operative Surveillance on Patient-Reported Outcomes in Patients With Stage II-III Soft Tissue Sarcoma of the Trunk and Extremities. https://clinicaltrials.gov/ct2/show/NCT04751409.

[17] Bae S., Karnon J., Crane G., Bessen T., Desai J., Crowe P., Neuhaus S. (2020). Cost-effectiveness analysis of imaging surveillance in stage II and III extremity soft tissue sarcoma: An Australian perspective. Cost Eff. Resour. Alloc..

[18] Royce T.J., Punglia R.S., Chen A.B., Patel S.A., Thornton K.A., Raut C.P., Baldini E.H. (2017). Cost–Effectiveness of Surveillance for Distant Recurrence in Extremity Soft Tissue Sarcoma. Ann. Surg. Oncol..

[19] Greenberg D.D., Crawford B. (2016). Surveillance Strategies for Sarcoma: Results of a Survey of Members of the Musculoskeletal Tumor Society. Sarcoma.

[20] Gerrand C.H., Billingham L.J., Woll P.J., Grimer R.J. (2007). Follow up after Primary Treatment of Soft Tissue Sarcoma: A Survey of Current Practice in the United Kingdom. Sarcoma.

[21] Goel A., Christy M.E., Virgo K.S., Kraybill W.G., Johnson F.E. (2004). Costs of follow-up after potentially curative treatment for extremity soft-tissue sarcoma. Int. J. Oncol..

[22] Acem I., Smit M.M., Verhoef C., van Houdt W.J., Haas R.L., van der Hage J.A., Grünhagen D.J., van de Sande M.A.J. (2021). Management of Soft Tissue Sarcomas in Extremities: Variation in Treatment Recommendations and Surveillance According to Specialty and Continent. Ann. Surg. Oncol..

[23] Gamboa A.C., Gronchi A., Cardona K. (2020). Soft-tissue sarcoma in adults: An update on the current state of histiotype-specific management in an era of personalized medicine. CA Cancer J. Clin..

[24] von Mehren M., Kane J.M., Agulnik M., Bui M.M., Carr-Ascher J., Choy E., Connelly M., Dry S., Ganjoo K.N., Gonzalez R.J. (2022). Soft Tissue Sarcoma, Version 2.2022, NCCN Clinical Practice Guidelines in Oncology. J. Natl. Compr. Canc. Netw..

[25] Swallow C.J., Strauss D.C., Bonvalot S., Rutkowski P., Desai A., Gladdy R.A., Gonzalez R., Gyorki D.E., Fairweather M., van Houdt W.J. (2021). Management of Primary Retroperitoneal Sarcoma (RPS) in the Adult: An Updated Consensus Approach from the Transatlantic Australasian RPS Working Group. Ann. Surg. Oncol..

[26] Callegaro D., Miceli R., Brunelli C., Colombo C., Sanfilippo R., Radaelli S., Casali P.G., Caraceni A., Gronchi A., Fiore M. (2015). Long-term morbidity after multivisceral resection for retroperitoneal sarcoma. Br. J. Surg..

[27] Raut C.P., Callegaro D., Miceli R., Barretta F., Rutkowski P., Blay J.-Y., Lahat G., Strauss D.C., Gonzalez R., Ahuja N. (2019). Predicting Survival in Patients Undergoing Resection for Locally Recurrent Retroperitoneal Sarcoma: A Study and Novel Nomogram from TARPSWG. Clin. Cancer Res..

[28] van Houdt W.J., Fiore M., Barretta F., Rutkowski P., Blay J.Y., Lahat G., Strauss D., Gonzalez R.J., Ahuja N., Grignani G. (2020). Patterns of recurrence and survival probability after second recurrence of retroperitoneal sarcoma: A study from TARPSWG. Cancer.

[29] Lin A.Y., Kotova S., Yanagawa J., Elbuluk O., Wang G., Kar N., Elashoff D., Grogan T., Cameron R.B., Singh A. (2015). Risk stratification of patients undergoing pulmonary metastasectomy for soft tissue and bone sarcomas. J. Thorac. Cardiovasc. Surg..

[30] Dossett L.A., Toloza E.M., Fontaine J., Robinson L.A., Reed D., Druta M., Letson D.G., Zager J.S., Gonzalez R.J. (2015). Outcomes and clinical predictors of improved survival in a patients undergoing pulmonary metastasectomy for sarcoma. J. Surg. Oncol..

[31] Smith R., Pak Y., Kraybill W., Kane J.M. (2009). Factors associated with actual long-term survival following soft tissue sarcoma pulmonary metastasectomy. Eur. J. Surg. Oncol..

[32] Chudgar N.P., Brennan M.F., Munhoz R.R., Bucciarelli P.R., Tan K.S., D’Angelo S.P., Bains M.S., Bott M., Huang J., Park B.J. (2017). Pulmonary metastasectomy with therapeutic intent for soft-tissue sarcoma. J. Thorac. Cardiovasc. Surg..

[33] Gutmann D.H., Ferner R.E., Listernick R.H., Korf B.R., Wolters P.L., Johnson K.J. (2017). Neurofibromatosis type 1. Nat. Rev. Dis. Prim..

[34] Evans D.G., Baser M.E., McGaughran J., Sharif S., Howard E., Moran A. (2002). Malignant peripheral nerve sheath tumours in neurofibromatosis 1. J. Med. Genet..

[35] Ahlawat S., Fayad L.M., Khan M.S., Bredella M.A., Harris G.J., Evans D.G., Farschtschi S., Jacobs M.A., Chhabra A., Salamon J.M. (2016). Current whole-body MRI applications in the neurofibromatoses: NF1, NF2, and schwannomatosis. Neurology..

[36] Ahlawat S., Blakeley J.O., Langmead S., Belzberg A.J., Fayad L.M. (2020). Current status and recommendations for imaging in neurofibromatosis type 1, neurofibromatosis type 2, and schwannomatosis. Skeletal Radiol..

[37] Tovmassian D., Abdul Razak M., London K. (2016). The Role of [(18)F]FDG-PET/CT in Predicting Malignant Transformation of Plexiform Neurofibromas in Neurofibromatosis-1. Int. J. Surg. Oncol..

[38] Mai P.L., Best A.F., Peters J.A., DeCastro R.M., Khincha P.P., Loud J.T., Bremer R.C., Rosenberg P.S., Savage S.A. (2016). Risks of first and subsequent cancers among TP53 mutation carriers in the National Cancer Institute Li-Fraumeni syndrome cohort. Cancer..

[39] Villani A., Tabori U., Schiffman J., Shlien A., Beyene J., Druker H., Novokmet A., Finlay J., Malkin D. (2011). Biochemical and imaging surveillance in germline TP53 mutation carriers with Li-Fraumeni syndrome: A prospective observational study. Lancet Oncol..

[40] Frebourg T., Bajalica Lagercrantz S., Oliveira C., Magenheim R., Evans D.G. (2020). Guidelines for the Li-Fraumeni and heritable TP53-related cancer syndromes. Eur. J. Hum. Genet..

[41] Bougeard G., Renaux-Petel M., Flaman J.M., Charbonnier C., Fermey P., Belotti M., Gauthier-Villars M., Stoppa-Lyonnet D., Consolino E., Brugières L. (2015). Revisiting Li-Fraumeni Syndrome From TP53 Mutation Carriers. J. Clin. Oncol..

[42] Tan M.C., Brennan M.F., Kuk D., Agaram N.P., Antonescu C.R., Qin L.X., Moraco N., Crago A.M., Singer S. (2016). Histology-based Classification Predicts Pattern of Recurrence and Improves Risk Stratification in Primary Retroperitoneal Sarcoma. Ann. Surg..

[43] Abbott A.M., Habermann E.B., Parsons H.M., Tuttle T., Al-Refaie W. (2012). Prognosis for primary retroperitoneal sarcoma survivors. Cancer.

[44] Callegaro D., Barretta F., Swallow C.J., Strauss D.C., Bonvalot S., Honorè C., Stoeckle E., van Coevorden F., Haas R., Rutkowski P. (2021). Longitudinal prognostication in retroperitoneal sarcoma survivors: Development and external validation of two dynamic nomograms. Eur. J. Cancer..

[45] Keung E.Z., Rajkot N., Torres K.E., Somaiah N., Hunt K.K., Feig B.W., Scally C.P., Ikoma N., Roland C.L. (2021). Evaluating the Impact of Surveillance Follow-Up Intervals in Patients Following Resection of Primary Well-Differentiated Liposarcoma of the Retroperitoneum. Ann. Surg. Oncol..

[46] Ikoma N., Roland C.L., Torres K.E., Chiang Y.J., Wang W.L., Somaiah N., Mann G.N., Hunt K.K., Cormier J.N., Feig B.W. (2018). Salvage Surgery for Recurrent Retroperitoneal Well-Differentiated Liposarcoma: Early Reoperation may not Provide Benefit. Ann. Surg. Oncol..

[47] Fiore M., Brunelli C., Miceli R., Manara M., Lenna S., Rampello N.N., Callegaro D., Colombo C., Radaelli S., Pasquali S. (2021). A Prospective Observational Study of Multivisceral Resection for Retroperitoneal Sarcoma: Clinical and Patient-Reported Outcomes 1 Year After Surgery. Ann. Surg. Oncol..

[48] Weaver R., O’Connor M., Carey Smith R., Sheppard D., Halkett G.K.B. (2021). “We’re on a Merry-Go-Round”: Reflections of Patients and Carers after Completing Treatment for Sarcoma. Curr. Oncol..

[49] Høeg B.L., Bidstrup P.E., Karlsen R.V., Friberg A.S., Albieri V., Dalton S.O., Saltbæk L., Andersen K.K., Horsboel T.A., Johansen C. (2019). Follow-up strategies following completion of primary cancer treatment in adult cancer survivors. Cochrane Database Syst. Rev..

[50] Bauml J.M., Troxel A., Epperson C.N., Cohen R.B., Schmitz K., Stricker C., Shulman L.N., Bradbury A., Mao J.J., Langer C.J. (2016). Scan-associated distress in lung cancer: Quantifying the impact of “scanxiety”. Lung. Cancer.

[51] Haber D.A., Velculescu V.E. (2014). Blood-based analyses of cancer: Circulating tumor cells and circulating tumor DNA. Cancer Discov..

[52] Coombs C.C., Dickherber T., Crompton B.D. (2020). Chasing ctDNA in Patients With Sarcoma. Am. Soc. Clin. Oncol. Educ. Book..

[53] Hemming M.L., Klega K.S., Rhoades J., Ha G., Acker K.E., Andersen J.L., Thai E., Nag A., Thorner A.R., Raut C.P. (2019). Detection of Circulating Tumor DNA in Patients With Leiomyosarcoma With Progressive Disease. JCO Precis Oncol..

